# Comparison of Perioperative Outcomes of Robotic-Assisted *vs* Laparoscopic Adrenalectomy for Pheochromocytoma: A Meta-Analysis

**DOI:** 10.3389/fonc.2021.724287

**Published:** 2021-09-16

**Authors:** Zhongyou Xia, Jinze Li, Lei Peng, Xiaoying Yang, Yulai Xu, Xianhui Li, Yunxiang Li, Zongping Zhang, Ji Wu

**Affiliations:** ^1^Department of Urology, Nanchong Central Hospital, The Second Clinical College, North Sichuan Medical College (University), Nanchong, China; ^2^Department of Urology, Institute of Urology, West China Hospital, Sichuan University, Chengdu, China; ^3^West China School of Medicine, Sichuan University, Chengdu, China; ^4^Blood Purification Center of Department of Nephrology, Nanchong Central Hospital, The Second Clinical College, North Sichuan Medical College, Nanchong, China

**Keywords:** pheochromocytoma, adrenalectomy, robotic, laparoscopic, meta-analysis

## Abstract

**Objective:**

To compare the efficacy and safety of robotic-assisted adrenalectomy (RA) and standard laparoscopic adrenalectomy (LA) for pheochromocytoma (PHEO).

**Methods:**

We systematically searched the Cochrane Library, PubMed, Embase, and Science databases for studies published through January 2021. Controlled trials on RA and LA for PHEOs were included. The meta-analysis was conducted with the Review Manager 5.4 software.

**Results:**

Four studies with 386 patients were included in the analysis. There were no significant differences in OT (WMD: 0.16; 95% CI: -28.50 to 28.82; I^2^ = 89%; P = 0.99), transfusion rate (OR: 0.70; 95% CI: 0.07 to 7.07; I^2^ = 64%; P = 0.77), conversion rate (OR: 0.44; 95% CI: 0.07 to 2.88; I^2^ = 0%; P = 0.39), complication rate (OR: 1.06; 95% CI: 0.62 to 1.82; I^2^ = 0%; P = 0.84) among patients undergoing RA and LA. However, compared with patients who underwent LA, patients who underwent RA had a shorter LOS (OR: -0.50; 95% CI: -0.55 to 0.45; I^2^ = 31%; P<0.01), less EBL (WMD: -0.85; 95% CI: -13.56 to -2.54; I^2^ = 44%; P<0.01), and fewer IHD (OR: 0.34; 95% CI: 0.17 to 0.70; I^2^ = 0%; P<0.01).

**Conclusion:**

The RA for pheochromocytoma achieve better outcomes over LA in terms of safety and efficacy.

## Introduction

Pheochromocytoma (PHEO) originates from the adrenal chromaffin tissue of the adrenal medulla (1). The majority of PHEO secretes catecholamines (epinephrine, norepinephrine, and dopamine), which are known to cause headaches, palpitations, and hyperhidrosis. However, this classical symptom triad is seen in only 25% of patients with PHEO (2, 3). Hypertension is the most common clinical symptom, with an incidence of approximately 80%–90% (2). In addition, approximately 12% of PHEO patients have cardiovascular complications, especially in patients with larger tumours at initial presentation (4).

Surgical resection of a tumour is an important management strategy to this day. Open adrenalectomy has been considered the gold standard technique for treating adrenal diseases, but the operation is often more traumatic for the patient and involves a large surgical incision. Since the 1990s, laparoscopic techniques have emerged as an alternative to open surgical approaches (5). Two recent meta-analyses have confirmed that laparoscopic adrenalectomy (LA) is associated with lower volume of bleeding, lower intraoperative hemodynamic instability (IHD) and better postoperative recovery when used in the treatment of PHEO (6, 7). However, because of the limited movement range of the instrument, physiological tremor magnification, and unclear two-dimensional images, it has been suggested that the risk of surgery is increased, especially for large PHEOs (8). The advent of robotic adrenalectomy (RA), however, has overcome some of these technical limitations through the introduction of three-dimensional operative viewing and increased manoeuvrability. An ever increasing number of studies have suggested that RA is a safe and feasible procedure for the resection of PHEO (8–10); nonetheless, Park et al. (11) found no major clinical advantages of robotic approaches compared to standard laparoscopy. Robotic approaches have also been suggested to increase cardiovascular events and even death in PHEO patients; the lack of tactile feedback during robot-assisted laparoscopic resections may lead to the increased release of the catecholamine hormone (9). Nonetheless, owing to the small sample size in these previous studies, it is difficult to obtain convincing evidence. Therefore, the safety and feasibility of RA and LA in the treatment of PHEO remain controversial.

We therefore performed a systematic review and meta-analysis to bridge this gap, to compare the safety and feasibility of RA and LA for the treatment of PHEO, and to better inform clinical practice.

## Methods

### Search Strategy

The article selection was in line with the Preferred Reporting Items for Systematic Reviews and Meta-Analysis (PRISMA) statement (12). We conducted a search in Embase (www.embase.com), Cochrane Library (www.cochranelibrary.com), PubMed (www.pubmed.ncbi.nlm.nih.gov), and Science databases up until January 2021. The following search terms were used: ‘laparoscopic OR laparoscopy’, ‘robot OR robotic-assisted’, ‘pheochromocytoma OR PHEO’, and ‘chromaffinoma’. The search strategies were tailored for different search engines. Further, the references in the relevant articles were also manually searched. The search was not limited by region or language. Two researchers (ZX and JL) independently conducted preliminary evaluations and data extraction from the literature.

### Inclusion and Exclusion Criteria

The inclusion criteria were as follows: (1) studies performed in adult (both male and female) patients with PHEO; (2) studies comparing RA to LA for PHEO; (3) at least one of the following outcome indicators: operating time (OT), estimated blood loss (EBL), transfusion rate (TR), conversion rate, IHD, overall complications, and length of stay (LOS); (4) study type was retrospective study, randomized controlled trial, cohort study, or case-control study.

The exclusion criteria were as follows: (1) patients diagnosed with paraganglioma; (2) paediatric patients; (3) studies without primary or sufficient data; (4) studies lacking a control group; and (5) case reports, reviews, and letters.

### Data Extraction

Two authors (ZX and JL) extracted the data independently. The following basic data were extracted from the enrolled studies: first author, publication date, country, study design, age of patients, and body mass index. Additionally, the surgical outcomes were extracted (including OT, EBL, IHD, conversion rate, TR, complications, and LOS).

### Quality Assessment

We used the New-Ottawa Scale (NOS) to assess the quality of our study. The range was 0 to 9. A score of ≥7 was considered high quality, the study score of 4–6 was considered medium quality, whereas a score of 0–4 was considered low quality. Three reviewers (ZX, JL, and LP) performed quality assessment of the included studies.

### Statistical Analysis

The meta-analysis were performed using Review Manager Version 5.4, software (The Cochrane Collaboration, Oxford, UK). The weighted mean difference (WMD) and odds ratio (OR) were calculated for continuous and dichotomous variables, respectively, with 95% confidence intervals (CIs). In addition, the heterogeneity between studies was evaluated using the χ^2^-test and inconsistency (I^2^) test. I^2^ >50% or P < 0.10, random-effects models were applied. Otherwise, fixed-effects models were used for the analyses. Finally, all p-values were two-tailed and the statistical significance level was set at 0.05. If continuous variables were expressed as median (interquartile range), then we converted them to mean ± standard deviation (13).

## Results

### Literature Search

**Figure 1:**
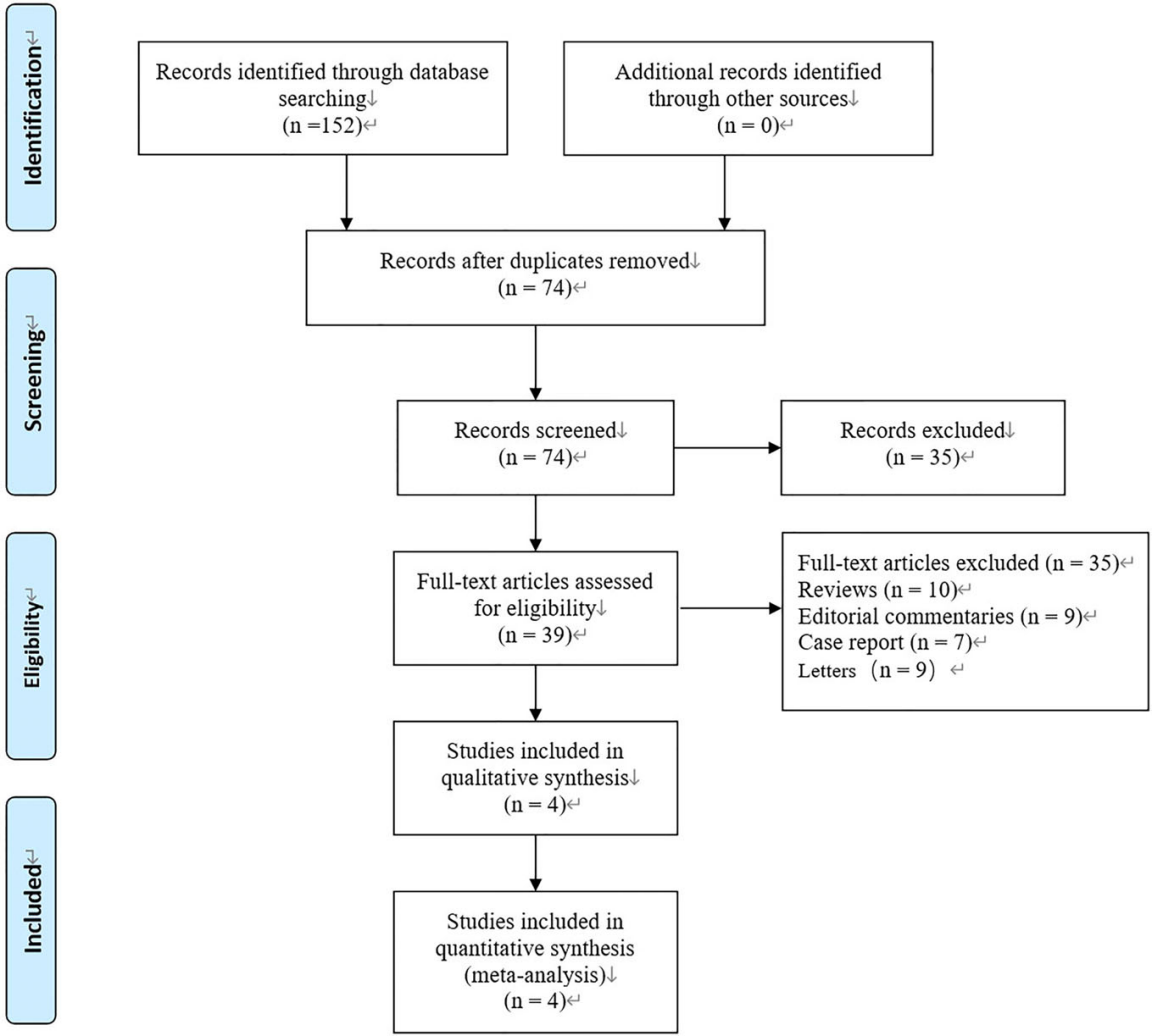
Flow diagram of studies identified, included, and excluded.

Initially, 152 published records were identified from the databases used (Embase, Cochrane Library, PubMed and others) according to our search strategy. Because of duplication, 78 articles were excluded, leaving 74 published records for inclusion in analysis. After reading the title and abstract further, 39 records were excluded (10 reviews, 9 editorial commentaries, 7 case reports, 9 letters). Four studies remained when reading the full text (8–10, 14) (**Figure 1**). These studies continued forward to our meta-analysis. Among the included studies, three were retrospective studies (9, 10, 14), and one study was a randomised controlled trial (8). The basic characteristics and quality evaluations of our study are shown in (**Table 1**).

**Table 1:** General characteristics and quality assessment of enrolled studies.

Study	Country	Design	Age(years)		BMI (kg/m^2^)		Quality score
			RA	LA	RA	LA	
Aliyev et al. (14)	America	Retrospective study	50.9±3.4	51.3±2.5	27.6±1.5	28.7±1.1	7
Fu et al. (7, 10)	China	Retrospective study	44±9.062	47.53±14.048	26.64±3.82	25.83±4.45	8
Fang et al. (9)	America	Retrospective study	55.9±15.4	46.2±17.9	24.5±4.9	29.8±6.5	7
Ma et al. (8)	China	Randomized controlled trial	44±14.4	47.7±17.4	21.8±3.3	22.9±3.3	7

LA, laparoscopic adrenalectomy; RA, robotic-assisted laparoscopic adrenalectomy; BMI, body mass index.

### Intraoperative Outcomes

#### Operation Time

**Figure 2:**
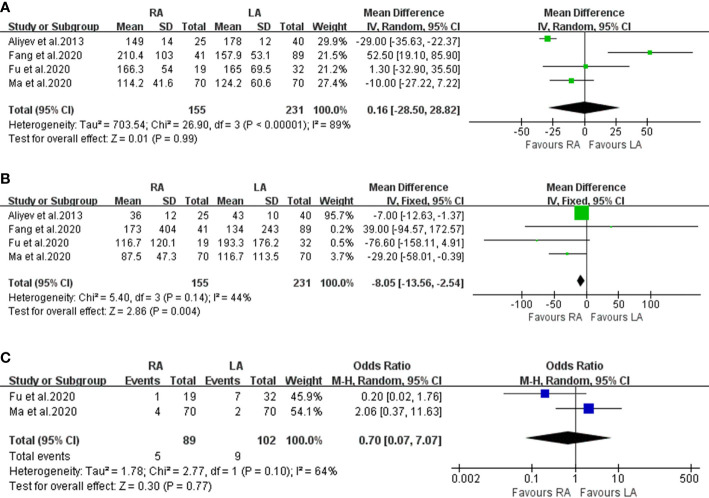
Forest plots of perioperative outcomes: **(A)** operating time, **(B)** estimated blood loss **(C)** transfusion rate.

Our meta-analysis included 386 patients. The RA group consisted of 155 patients and the LA group had 231 patients that met our criteria for OT (8–10, 14). There was no significant difference in OT between the two groups (WMD: 0.16; 95% CI: -28.50 to 28.82; I^2^ = 89%; P = 0.99) (**Figure 2A**).

#### Estimated Blood Loss and Transfusion Rate

All four articles reported EBL, and the relevant data were extracted from the studies (n= 386 patients, RA=155 *vs* LA=231) (8–10, 14), (**Figure 2B**). The combined results showed that the difference in EBL was statistically significant between RA and LA (WMD: -0.85;95% CI: -13.56 to -2.54; I^2^ = 44%; P < 0.01). Compared to the LA group, there was a lower volume of blood loss in the RA group. However, only two trials (which included 191 patients) evaluated TR (**Figure 2C**). Significant heterogeneity existed in the pooled data, and no significant difference was found between the two groups (OR: 0.70; 95% CI: 0.07 to 7.07; I^2^ = 64%; P = 0.77).

#### Conversion Rate

**Figure 3:**
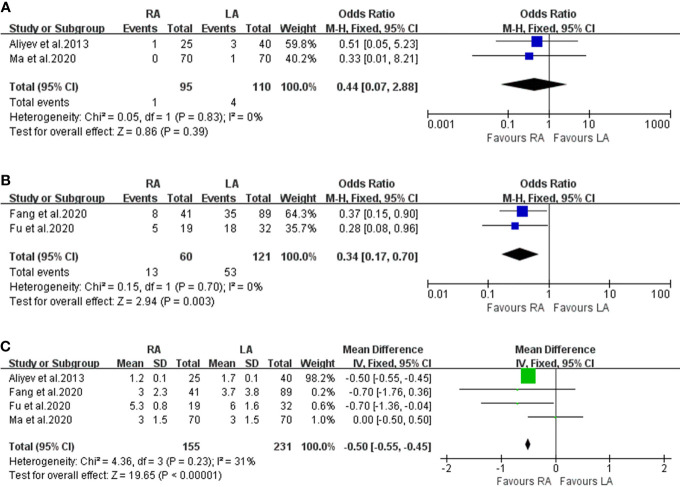
Forest plots of perioperative outcomes: **(A)** conversion rate, **(B)** intraoperative hemodynamic instability, **(C)** length of hospital stay.

The conversion rate data were obtained from two studies (8, 14) (**Figure 3A**). There was no significant difference between the RA and LA groups (OR 0.44; 95% CI, 0.07 to 2.88; I^2^ = 0%; P = 0.39).

#### Intraoperative Haemodynamic Instability

Two articles were analysed in our meta-analysis for IHD. A total of 181 patients were included, where 60 underwent RA and 121 underwent LA (**Figure 3B**). Compared to the LA group, fewer patients in the RA group presented with IHD (OR: 0.34; 95% CI: 0.17 to 0.70; I^2^ = 0%; P < 0.01).

### Postoperative Outcomes

#### Length of Hospital Stay

Data on LOS were recorded in 4 studies involving 386 patients (**Figure 3C**) (8–10, 14). The merged results identified that RA had a shorter postoperative LOS than LA (OR: -0.50; 95% CI: -0.55 to 0.45; I^2^ = 31%; P<0.01).

#### Complication Rate

**Figure 4:**
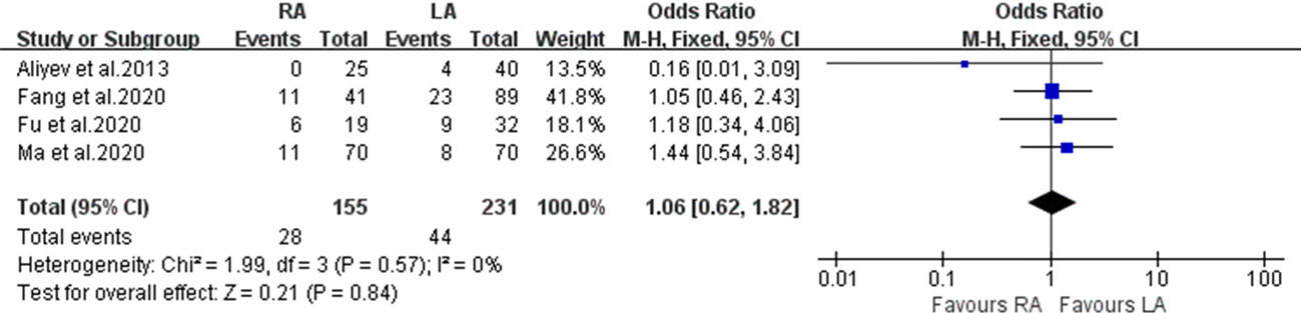
Forest plot for complication rate.

In the statistical analysis, 386 patients had ‘overall complications’ data documented (RA 155 and LA 231) (**Figure 4**). No significant difference between the RA and LA groups was found (OR, 1.06; 95% CI, 0.62 to 1.82; I^2^ = 0%; P = 0.84).

## Discussion

Pheochromocytoma (PHEO) treatment has always been challenging for urologists; their rich vascularity and paroxysmal catecholamine secretions indicate that adrenalectomy may cause adverse health outcomes for patients. Because PHEO can secrete paroxysmal catecholamines and have rich vascularity, adrenalectomy has always been challenging for urologists. Since laparoscopic adrenalectomy has been used for adrenal tumour removal (including PHEO) since 1992, its efficacy and safety have been well documented (15). In the current era of robots, numerous endocrine surgery teams now advocate the use of robot-assisted laparoscopic adrenalectomy for PHEO (16). However, there is no consensus regarding the true benefit of robotic surgery over conventional laparoscopy in the treatment of PHEO.

To the best of our knowledge, this is the first study focusing on robotic adrenalectomy (RA) for the treatment and outcomes of pheochromocytoma in adult patients in comparison with laparoscopic adrenalectomy (LA). We systematically reviewed the literature on the two surgery types for PHEO and identified 4 eligible articles, which included 386 patients. However, only one randomised clinical trial (RCT) comparing RA to LA was involved (8). In our meta-analysis, no significant differences were detected between the RA and LA groups in terms of demographic characteristics. Our study showed that the RA group achieved better outcomes in terms of estimated blood loss (EBL), transfusion rate (TR), intraoperative hemodynamic instability (IHD), and length of hospital stay (LOS) than the LA group. However, the RA group was not significantly different from LA in terms of operation time (OT), conversion rate, and overall complications.

Limited studies reported the operating time (OT) and surgical outcomes showed that there were no significant differences between the two groups with high heterogeneity. Our finding is consistent with that of a previous study where operative time was also similar between the two groups (10). However, other studies show varied results; in the study by Pineda-Solís et al. (17), 30 RA and 30 traditional LA showed an increase in operative time in the robotic system, conversely Ma et al. (8) and Silay et al. (18) reported that the total operative time was shorter in the robotic group. This could be explained by the progressive improvements in laparoscopic robotic technology over time, allowing it to become as effective in surgical path decisions for similar sized tumours. However, because of the lack of sufficient data, subgroup analysis was not performed here. Our high heterogeneity may be explained by the initial tumour size, robot type installed, and surgical path decisions as was also suggested by the previous authors.

Blood loss has always been the major focus of clinicians. In our meta-analysis the EBL of RA were found to be lower than that of LA. In our view, using joint instruments, a three-dimensional view, and a more stable camera platform, robotics can speed up adrenalectomy and account for less intraoperative bleeding during surgery. However, this result is in contradiction to the data reported in two previous studies. Fang et al. (9) performed a retrospective study where they reported no significant difference between the two groups (RV or LV) for EBL. Similar results were also obtained by Aliyev et al. (14). The data from the two studies show that the RA group had a larger initial tumour size and higher patient BMI, which restricts the advantages of the robot. In addition, we compared the TR of the two procedures (RA *versus* LA). Although there was no statistical difference in the blood TR between the two groups, an exciting finding in our analysis was that the RA group had a lower TR than the LA group. This could be explained by the robot’s flexible arm, magnified high-definition 3D stereo vision that can more accurately display deep anatomy and separation, and intraoperative bleeding can be more easily detected and controlled. This is especially true for large PHEO size, which has a rich blood supply.

According to our present study, only two articles reported conversion rate, and no significant difference between the RA and LA groups was found. Economopoulos et al. (19) performed a meta-analysis about the conversion rate between RA and LA, the results were similar to those of our meta-analysis. A retrospective study reported that conversion to an open procedure in 3 patients in the LA group and 1 patient in the RA group (14). Similarly, Ma et al. (8) conducted a randomised controlled trial that included 140 patients, and a slightly higher conversion rate was found in the LA group (LA 1.4% *vs.* RA 0%). A higher conversion rate in the LA group was thought to be caused by more bleeding and tumour adherence to the surrounding tissues (8). Moreover, laparoscopic instruments can often be inferior to robots in terms of range of motion and image quality.

Controlling IHD is challenging for surgeons performing PHEO resections. Of note, the incidence of IHD during PHEO resection is between 17% and 83% according to the literature (20), which is also consistent with our results. In our meta-analysis, pooled data revealed that RA provided significantly lower IHD than LA, with no heterogeneity (p < 0.05). According to Fang et al., higher IHD is attributed to surgeon preference for the tactile feedback of laparoscopy, which stimulates the pheochromocytoma to release large amounts of catecholamines (9). However, robotic surgery is more accurate and less vibratory, thereby reducing the possibility of tumours being squeezed during surgery; therefore, intraoperative blood pressure fluctuations will be minimal (10). Moreover, the IHD is still low in robotic adrenalectomy for large PHEO (size ≥6 cm) (10). Whether there is an effect on IHD in terms of surgical path, Jiang et al. found no significant difference in IHD between the transperitoneal and retroperitoneal approaches (21). Through multi-factor analysis, they found that retroperitoneal adrenalectomy increased the incidence of mean arterial pressure of <60 mmHg compared with the transperitoneal path (22).

In the present study, we found that RA can significantly reduce the LOS. This is similar to other previous studies (9, 10, 14). We found that the following factors may be related to the results of the study: first, robotic surgery had little effect on the IHD of the patients; therefore, the vital signs of the patients were stable after the operation; second, the pain score on postoperative day was lower, and less discomfort was reported in the robotic group; and third, there could be individual surgeon bias and hospitalisation costs involved. However, the studies of You et al. and Ma et al. showed no overt difference in mean LOS between the two groups (8, 23).

In terms of the overall complication rate, our study showed no significant difference between the RA and LA groups. Postoperative hypertension and hypotension are major post-adrenalectomy complications (9). Chen et al. reported that a tumour size of > 6 cm was an independent risk factor associated with increased perioperative complications (24). Therefore, our results may be explained by the fact that stable IHD may be associated with the use of α-adrenergic receptor blockers preoperatively (to control blood pressure), surgeons often choose the surgical approach they are familiar with, and larger PHEOs were in the RA group.

Finally, the cost of RA is a road-block to its widespread use, especially in developing countries. Several studies have shown that the cost of RA is higher than that of LA (8, 9, 25). A previous study reported that RA adds up to $950 to the LA (25). Surprisingly, Ma et al. found that the cost of RA was nearly twice that of LA ($8869.9 *vs.* $4,721.8, P < 0.01) (8). By contrast, Feng et al. found that both procedures had similar costs, which may be related to limiting extraneous robotic instruments and surgical team experience (26). Notably, Brunaud et al. showed that increasing the number of robot surgeries per year and the depreciation of robot systems were effective in reducing costs (27). We believe that RA could become more affordable in high-volume robotic surgery centres in the future (19).

As the first meta-analysis to directly compare the perioperative outcomes of RA and LA in patients with PHEO, our study is of high clinical interest. However, there were some limitations to this study. First, only one RCT was included, and the sample size overall was small. Larger RCTs are needed to further validate our findings. Second, the inclusion criteria did not include two-sided PHEOs or surgical paths in our study. Finally, some outcomes were heterogeneous, including OT and TR. Limited data restricted the ability to perform subgroup analysis and sensitivity analysis; therefore, some results in our meta-analysis should be interpreted with caution.

## Conclusions

According to our current systematic review and comprehensive meta-analysis, the RA for PHEO can yield better outcomes than LA in terms of safety and efficacy. Future studies utilising larger randomised controlled trials comparing RA and LA for PHEO may provide better evidence.

## Data Availability Statement

The original contributions presented in the study are included in the article/supplementary material. Further inquiries can be directed to the corresponding author.

## Author Contributions

ZZ, YL and JW: conception and design. XZ, JL, XY, and XL: acquisition of data and critical revision of the manuscript for important intellectual content. ZX, JL, XY, LP and XL: analysis and interpretation of data. ZX and JL: drafting of the manuscript. JW: supervision. All authors contributed to the article and approved the submitted version.

## Funding

This article was funded by the special project of Nanchong City School Cooperation (NSMC20170457) and by the Technology Department Project of Sichuan Science (2020YFS0320).

## Conflict of Interest

The authors declare that the research was conducted in the absence of any commercial or financial relationships that could be construed as a potential conflict of interest.

## Publisher’s Note

All claims expressed in this article are solely those of the authors and do not necessarily represent those of their affiliated organizations, or those of the publisher, the editors and the reviewers. Any product that may be evaluated in this article, or claim that may be made by its manufacturer, is not guaranteed or endorsed by the publisher.
